# Exposure to Polyfluoroalkyl Chemicals and Cholesterol, Body Weight, and Insulin Resistance in the General U.S. Population

**DOI:** 10.1289/ehp.0901165

**Published:** 2009-11-02

**Authors:** Jessica W. Nelson, Elizabeth E. Hatch, Thomas F. Webster

**Affiliations:** 1 Department of Environmental Health and; 2 Department of Epidemiology, Boston University School of Public Health, Boston, Massachusetts, USA

**Keywords:** body mass index, cholesterol, insulin resistance, National Health and Nutrition Examination Survey, perfluorononanoic acid, perfluorohexane sulfonic acid, perfluorooctane sulfonic acid, perfluorooctanoic acid, polyfluoroalkyl chemicals, waist circumference

## Abstract

**Background:**

Polyfluoroalkyl chemicals (PFCs) are used commonly in commercial applications and are detected in humans and the environment worldwide. Concern has been raised that they may disrupt lipid and weight regulation.

**Objectives:**

We investigated the relationship between PFC serum concentrations and lipid and weight outcomes in a large publicly available data set.

**Methods:**

We analyzed data from the 2003–2004 National Health and Nutrition Examination Survey (NHANES) for participants 12–80 years of age. Using linear regression to control for covariates, we studied the association between serum concentrations of perfluorooctanoic acid (PFOA), perfluorononanoic acid (PFNA), perfluorooctane sulfonic acid (PFOS), and perfluorohexane sulfonic acid (PFHxS) and measures of cholesterol, body size, and insulin resistance.

**Results:**

We observed a positive association between concentrations of PFOS, PFOA, and PFNA and total and non-high-density cholesterol. We found the opposite for PFHxS. Those in the highest quartile of PFOS exposure had total cholesterol levels 13.4 mg/dL [95% confidence interval (CI), 3.8–23.0] higher than those in the lowest quartile. For PFOA, PFNA, and PFHxS, effect estimates were 9.8 (95% CI, −0.2 to 19.7), 13.9 (95% CI, 1.9–25.9), and −7.0 (95% CI, −13.2 to −0.8), respectively. A similar pattern emerged when exposures were modeled continuously. We saw little evidence of a consistent association with body size or insulin resistance.

**Conclusions:**

This exploratory cross-sectional study is consistent with other epidemiologic studies in finding a positive association between PFOS and PFOA and cholesterol, despite much lower exposures in NHANES. Results for PFNA and PFHxS are novel, emphasizing the need to study PFCs other than PFOS and PFOA.

Polyfluoroalkyl chemicals (PFCs) are a class of highly stable compounds used widely in commercial and industrial applications as surfactants, paper and textile coatings, and food packaging (Calafat et al. 2007). Numerous chemicals belong to this class, including the products used industrially, by-products of manufacturing, and degradation products. They are composed of a fluorinated carbon backbone of varying length terminated by a carboxylate or sulfonate functional group. This amphipathic structure provides the properties of water and oil repellency and stain resistance (Conder et al. 2008). The perfluorinated carboxylates include perfluorooctanoic acid (PFOA) and perfluorononanoic acid (PFNA), and the perfluorinated sulfonates include perfluorooctane sulfonic acid (PFOS) and perfluorohexane sulfonic acid (PFHxS).

Biomonitoring studies have documented human exposure to PFCs, both in occupationally exposed cohorts (Costa et al. 2009; Olsen and Zobel 2007; Sakr et al. 2007a) and in the general population (Apelberg et al. 2007; Calafat et al. 2007; Fei et al. 2007). Although the major sources of human exposure are poorly known, possibilities include diet (either directly from food or migration from food packaging), drinking water, and house dust (reviewed in Lau et al. 2007).

Once taken into the human body, PFCs are slowly eliminated and are not known to undergo biotransformation (Lau et al. 2007). They bioaccumulate, but not in lipid as do many other persistent organic pollutants. Instead, they bind to proteins in the liver and serum (Conder et al. 2008). Mean serum half-lives in humans are estimated as 5.4 years for PFOS and 3.8 years for PFOA (Olsen et al. 2007). Shorter-chain compounds are generally assumed to have shorter half-lives, although PFHxS is an exception, with an estimated mean half-life of 8.5 years (Olsen et al. 2007). The half-life for PFNA in humans has not been estimated.

Various adverse health effects have been observed in animal studies of PFOS and PFOA, including tumors in certain organs and developmental delays (Biegel et al. 2001; White et al. 2007). The structural resemblance of PFCs to fatty acids and the discovery that they bind to peroxisome proliferator-activated receptors (PPARs), nuclear receptors that play a key role in lipid metabolism and adipogenesis, have raised the concern that PFCs may disrupt lipid and weight regulation. Indeed, among the early reported health effects in animal studies that administered high PFC doses was hypolipidemia (Seacat et al. 2002). However, several studies in humans suggest that exposure to PFOA, and possibly to PFOS, may be associated with increased cholesterol in humans (C8 Science Panel 2008; Costa et al. 2009; Sakr et al. 2007a). The evidence for an association between PFC exposure and body size and insulin resistance is much weaker (Lin et al. 2009).

The rising prevalence of the metabolic syndrome, which includes obesity, dyslipidemia, and insulin resistance, is of increasing public health concern in the United States and globally and is linked closely with coronary heart disease and related disorders (Ramos and Olden 2008). Although changes in diet and lifestyle are undoubtedly important factors in this trend, there is growing interest in the hypothesis that endocrine-disrupting chemicals may be playing a role (Grun and Blumberg 2009).

This exploratory, cross-sectional epidemiologic study investigated the relationship between exposure to four PFCs, including two compounds that have been little studied in humans, and cholesterol levels, obesity, and insulin resistance.

## Materials and Methods

### Study population

The National Health and Nutrition Examination Survey (NHANES) is an ongoing survey of the civilian noninstitutionalized U.S. population conducted by the Centers for Disease Control and Prevention (CDC) that gathers data on dietary and health factors. Participants are selected using a complex multistage probability sampling design and come to a mobile examination center for a physical examination and to provide blood and urine samples. Various questionnaires are administered by trained interviewers (CDC 2009). The survey also includes biomonitoring for different environmental chemicals, including PFCs, of a random one-third subsample of participants by the National Center for Environmental Health (NCEH). NHANES obtained informed consent from all participants.

### PFC concentrations

PFCs were measured in serum of participants ≥ 12 years of age by the NCEH using automated solid-phase extraction coupled to isotope dilution/high-performance liquid chromatography/tandem mass spectrometry; details of laboratory methods are available elsewhere (Calafat et al. 2007). Our study examined the four PFCs detected in > 98% of people: PFOS, PFOA, PFHxS, and PFNA. The other eight PFCs measured were detected in < 28% of people. Values below the limit of detection (LOD) were reported by NHANES as the LOD divided by the square root of 2.

### Outcomes

Several cholesterol measures are commonly used in clinical and epidemiologic studies. Cholesterol is carried in plasma within different lipoproteins, including low-density lipoproteins (LDLs) and very low-density lipoproteins (VLDLs) which carry cholesterol to peripheral tissues and are considered “bad” cholesterol, and high-density lipoproteins (HDLs) which transport cholesterol back to the liver for excretion and are considered “good” cholesterol. LDL carries around 70% of total plasma cholesterol, and HDL 20−30% (Tietz et al. 2006). Total cholesterol (TC) is the sum of the cholesterol content of LDL, HDL, and VLDL. The non-HDL cholesterol fraction, which includes LDL and VLDL cholesterol, has been shown to be a better predictor of risk of coronary heart disease than LDL alone (Liu et al. 2006).

We studied TC, HDL, non-HDL, and LDL. TC and HDL were measured by NHANES directly in serum of all participants; TC was measured enzymatically through coupled reactions that hydrolyze cholesteryl esters, and HDL after the precipitation of apolipoprotein B lipoproteins with a blocking agent (CDC 2007b). We calculated non-HDL by subtracting HDL from TC. LDL was available only for the subsample of fasting participants and was not measured directly in serum, but estimated by NHANES using the widely accepted Friedewald formula (CDC 2007c).

Body size outcomes considered include body mass index (BMI): weight (kilograms) divided by height (meters squared), and waist circumference (WC; centimeters). Weight, height, and WC were measured during the examination using standard protocols (CDC 2007d). To assess insulin resistance, we studied homeostatic model assessment (HOMA), used in epidemiologic studies as a simple, inexpensive, and reliable alternative to more complicated methods (Bonora et al. 2000). We calculated HOMA using the method of Matthews et al. (1985): HOMA = [fasting insulin (μU/mL) × fasting glucose (mmol/L)]/22.5. Plasma insulin and glucose were measured enzymatically by NHANES in the fasting subsample of participants (CDC 2007a).

### Covariates

NHANES collected data on potential confounding variables through questionnaires. Because we had a large sample size, our models included *a priori* a number of covariates that are important predictors of cholesterol and body weight: age, sex, race/ethnicity, socioeconomic status (SES, a dichotomous indicator that combined income, education, and food insecurity to minimize missing data), saturated fat intake (tertiles, as percentage of total caloric intake), exercise (performed moderate or vigorous physical activity in the preceding 30 days), and time in front of a TV or computer (categories of hours per day in the preceding 30 days). For those ≥ 20 years of age, we also included alcohol consumption (categories of drinks per week), smoking, and, for women, parity. For the cholesterol analyses, we included continuous BMI as a covariate, and tested for confounding by continuous serum albumin. See Supplemental Material, Table 1, available online (doi:10.1289/ehp.0901165.S1 via http://dx.doi.org/) for details on covariates.

### Statistical analysis

We performed regression analyses in sex and age (12–19, 20–59, 60–80 years) subgroups for each PFC separately. When results showed similar trends by age and sex, we combined groups. Cholesterol and weight outcomes were analyzed as continuous variables; HOMA was log-transformed as it was log-normally distributed. For the main analysis, exposure was modeled in quartiles of PFC concentration, with quartiles formed in the population overall and separately for the age/sex group used in the analysis. We present effect estimates for each quartile compared with the reference group (the first quartile) and their corresponding 95% confidence intervals (CIs). Tests for trend in the quartile analyses were performed by treating PFC category as a linear predictor in the models.

In addition, for cholesterol outcomes in adults, we performed a sensitivity analysis that modeled exposure as a continuous predictor. We identified influential points and outliers by examining studentized residuals, predicted values, and scatter plots, and excluded them from the analysis if they changed the effect estimates by ≥ 5%.

All analyses excluded those > 80 years of age, pregnant, breast-feeding, taking insulin, or undergoing dialysis. Cholesterol analyses also excluded those who reported current use of cholesterol-lowering medications in the blood pressure portion of the questionnaire or who were missing this variable. See Supplemental Material, Figure 1 (doi:10.1289/ehp.0901165.S1), for the number of people in each exclusion group. Covariates described above were used in all models for which they were available, depending on age group and sex.

To perform analyses, we used the SAS version 9.1 Proc SURVEYREG (SAS Institute Inc., Cary, NC) procedure, which takes into account possible correlation between the strata and clusters by which NHANES samples the population. Models were adjusted for relevant covariates instead of using NHANES sampling weights; this adjustment is regarded as a good compromise between efficiency and bias (Korn and Graubard 1991).

## Results

PFC concentrations were available for 2,094 participants of the original subsample of 2,368 people. PFOS levels were an order of magnitude higher than the other PFCs, with a median of 19.9 μg/L serum compared with 3.8 μg/L for PFOA. Similar to results in the same data set reported by Calafat et al. (2007), concentrations were higher in males compared with females, non-Hispanic whites compared with Mexican Americans and non-Hispanic blacks, and people of higher SES compared with those of lower SES. There were no striking concentration differences by age. The four PFCs were log-normally distributed and were moderately correlated with one another. PFOA and PFOS were most strongly correlated, with a Spearman correlation coefficient of 0.65; PFHxS and PFNA were the least correlated at 0.12. Cholesterol, body weight, and insulin resistance outcomes varied with age, sex, and race/ethnicity and were correlated with one another in predictable ways.

**Figure 1:**
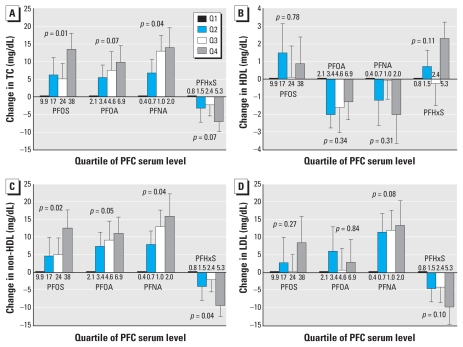
Differences in cholesterol levels, persons 20–80 years of age, with increasing quartile of PFC exposure. (*A*) Change in TC (*n* = 860), (*B*) change in HDL (*n* = 860), (*C*) change in non-HDL (*n* = 860), and (*D*) change in LDL (*n* = 416). All models control for age, sex, race/ethnicity, SES, saturated fat intake, exercise, time in front of a TV or computer, alcohol consumption, smoking, and BMI. Median PFC levels (micrograms per liter) for each quartile are shown below/above the bar. Error bars represent SEs of the effect estimates (i.e., the difference between the quartile and the reference group), and *p*-values for trend are presented; 95% CIs for each effect estimate are available in Supplemental Material, Table 3 (doi:10.1289/ehp.0901165.S1).

The number of participants in each analysis depended on the outcome and missing data. We present results for the cholesterol analyses among adults (20–80 years of age) in Tables 1 and 2 and Figure 1. Supplemental Material, Figure 1 (doi:10.1289/ehp.0901165.S1), illustrates how we arrived at our final sample size, which does not include 12- to 19-year-olds (*n* = 640). Of adults with PFC and cholesterol measures (*n* = 1,310), we excluded the 20% who reported using cholesterol-lowering medications and the 3% who were missing this variable. None of the covariates were missing in > 9% of people. Table 1 shows the distribution of outcomes and PFC concentrations in this subpopulation, including PFC range and number of people in each quartile. In all cases, the PFC range in the fourth quartile is much wider than in the other three quartiles. Supplemental Material, Table 1 (doi:10.1289/ehp.0901165.S1), provides information on the distribution of covariates.

### Cholesterol

Figure 1 presents the adjusted associations between the four cholesterol measures and PFC serum concentrations for adults [Supplemental Material, Table 2 (doi:10.1289/ehp.0901165.S1], presents crude associations). We omitted 12- to 19- year-olds because no data were available for two important covariates, alcohol and smoking. See Supplemental Material, Table 3, for results stratified by age (including 12- to 19-year-olds) and sex.

We found a positive association between TC and PFOS, PFOA, and PFNA concentrations (Figure 1A). Adults in the highest PFOS quartile had TC levels 13.4 mg/dL (95% CI, 3.8–23.0) higher than those in the lowest quartile. For PFOA, there was a 9.8‐mg/dL (95% CI, −0.2 to 19.7) increase, and for PFNA, a 13.9-mg/dL (95% CI, 1.9–25.9) increase. TC appeared to increase linearly across the quartiles of PFC exposure, particularly for PFNA (*p*-value for trend = 0.04). When examined in age and sex subgroups, results were similar, with associations of greater magnitude among persons 60–80 years of age. Associations were fewer and of smaller magnitude among 12- to 19-year-olds. In contrast, results for PFHxS indicated an inverse trend among adults (*p*-value for trend = 0.07). Those in the top PFHxS quartile had TC levels that were lower than those in the lowest quartile by −7.0 mg/dL (95% CI, −13.2 to −0.8). The same pattern held in the female age subgroups in particular.

We found fewer consistent trends in the HDL analyses. We observed differences by age and sex; results for all adults (Figure 1B) may mask these findings in some cases [see Supplemental Material, Table 3 (doi:10.1289/ehp.0901165.S1)]. PFOA and PFOS were associated with higher HDL in adolescent girls [effect estimates for the top quartile compared with lowest of 4.3 (95% CI, 0.1 to 8.5) and 3.7 (95% CI, −0.5 to 7.9), respectively], with some evidence of the opposite in the older age group [in males 60–80 years of age, effect estimate for the top PFOA quartile compared with the lowest of −8.7 (95% CI, −16.3 to −1.1)]. No meaningful associations were observed between PFNA and PFHxS concentration and HDL.

Results for non-HDL were similar to those for TC, as would be expected, because the non-HDL fraction makes up 70–80% of TC (Figure 1C). The magnitude of effect increased slightly for PFNA and PFHxS. LDL results (Figure 1D) should mirror those for non-HDL; however, the sample size for LDL analyses was half as large. We found a somewhat similar pattern for PFNA and PFHxS, but no association with PFOA and PFOS concentration.

We repeated all cholesterol models and adjusted for albumin. Results were substantively the same as those presented above (data not shown). Results were similar as well in models that considered PFC concentration as a continuous predictor (Table 2). PFOS, PFOA, and PFNA were all positively associated with TC and non-HDL (effect estimates were statistically significant for PFOS and PFOA). The opposite was seen for PFHxS, which was negatively associated with TC, non-HDL, and LDL.

In addition, we performed several sensitivity analyses that also had no qualitative effect on results from the quartile analysis: the inclusion of adults missing data on use of cholesterol-lowering medication, the inclusion of all adults (even those who reported taking medications), the exclusion of points identified as outliers in the continuous models from Table 2, and use of NHANES sampling weights.

### Body weight

We found fewer meaningful associations between body weight and PFC concentrations [see Supplemental Material, Table 3 (doi:10.1289/ehp.0901165.S1)]. The strongest effects were seen with PFOS among males. In males 12–19 and 20–59 years of age, BMI decreased with increasing PFOS exposure. Teenage boys in the highest PFOS quartile had BMIs that were 2.8 points (95% CI, −4.1 to −1.4) lower than those in the lowest quartile (*p*-value for trend = 0.004). In men 60–80 years of age, on the other hand, increasing PFOS exposure was associated with increased BMI [effect estimate for the top quartile compared with lowest of 1.6 (95% CI, 0.14–3.0)]. We did not see evidence of a relationship in the female age groups. Results for the other PFCs were less consistent, and those for WC were similar to BMI.

### HOMA

On the whole, we found no association between PFC concentrations and HOMA. Although there were isolated suggestive trends, such as a significant positive trend with PFNA in adult females and a negative one with PFHxS in adolescent females, effects were not consistent [see Supplemental Material, Table 3 (doi:10.1289/ehp.0901165.S1)].

## Discussion

This exploratory study examined associations between serum concentrations of four PFCs and cholesterol levels, body size, and insulin resistance in a sample of the general U.S. population. Most striking were the findings for TC and non-HDL. These outcomes were positively associated with PFOS, PFOA, and PFNA and negatively associated with PFHxS after controlling for numerous covariates in categorical and continuous models. The LDL analyses were more limited by study size, but revealed similar trends for PFHxS and PFNA, although of less consistency and magnitude. No strong trends emerged in the HDL analyses. These results suggest that exposure to background levels of certain PFCs may exert effects on the non-HDL fraction of cholesterol. We did not find consistent associations between PFCs and BMI, WC, or HOMA.

### Previous studies in humans

Studies of the association between cholesterol levels and PFCs are found primarily in the occupational health literature. Although results are not entirely consistent, the general trend is one of positive associations between PFOA concentration and cholesterol levels. Results for PFOS are less clear, as it has been less studied. Sakr et al. (2007a, 2007b) studied a large cohort of DuPont workers (*n* = 454 for a longitudinal study and 1,025 for a cross-sectional study). In both, PFOA was positively associated with TC but not with HDL. A positive association was observed with LDL in the cross-sectional study only. When restricted to those not taking cholesterol-lowering medications, the magnitude of effect in the cross-sectional study increased. A study of a smaller group of Italian workers (*n* = 53), which included a substudy excluding those being treated for hyperlipidemia, found a similar positive association between TC and PFOA (Costa et al. 2009). Findings from studies of workers at different 3M Company locations are more mixed. The most recent study conducted by Olsen and Zobel (2007) did not find evidence of an association between serum PFOA and TC or LDL among 506 employees at three facilities. An earlier study of the same workers at two of those locations, which did not adjust for use of cholesterol-lowering medications, found a positive association between serum PFOS and PFOA and TC in a cross-sectional analysis (*n* = 421) and PFOA in a longitudinal analysis (*n* = 174) (Olsen et al. 2003).

Exposure levels in these workers are much higher than in NHANES participants. Median serum concentrations in the 3M cohort were 1,100 μg/L for PFOA and 720 μg/L for PFOS (Olsen and Zobel 2007). The mean PFOA level was 4,300 μg/L in the DuPont studies (Sakr et al. 2007a); the median was 3,890 μg/L in 2007 measurements from the Italian cohort (Costa et al. 2009). In comparison, median serum concentrations in NHANES were 4 and 20 μg/L for PFOA and PFOS, respectively.

Two studies have also been conducted on PFCs and cholesterol outcomes in communities surrounding a DuPont plant that have much higher PFOA exposures than the general population. Emmett et al. (2006) examined PFOA concentrations among 371 residents of a water district area bordering the plant. Although the study found no association between PFOA and TC, the analyses neither controlled for possible confounders nor excluded people on cholesterol-lowering medications. The C8 Health Project, a much larger study conducted in relation to a legal case, has released preliminary, non-peer-reviewed findings from its analysis of 46,294 people living in six water districts near the plant (C8 Science Panel 2008). The study, which excluded those on cholesterol medications and controlled for confounding, found significant positive associations between PFOA and PFOS concentrations and TC and LDL.

A recent study using NHANES data examined the relationship between PFCs and components of the metabolic syndrome in 1999–2000 and 2003–2004 participants (Lin et al. 2009). It is difficult to compare our study with these results, as the authors examined an additional 2 years of data, did not report results for TC, non-HDL, or LDL, and conducted logistic regression analyses for two of the outcomes. They found a significant positive association between HOMA and PFOS concentrations in adults, similar to the direction of association we observed (although, in our study, the trend did not come close to statistical significance). PFNA concentrations were found to have a protective effect on the odds of having low HDL in adolescents and adults, with the opposite seen for PFOS in adults. Our study did not observe these relationships with HDL as a continuous outcome. Finally, the authors found that higher PFHxS, PFOA, and PFOS concentrations in adolescents were associated with decreased WC, findings we also observed for PFOS.

There have been few studies of nondevelopmental PFC exposure and body size. A cross-sectional study of 3M workers found BMI to be slightly higher in the highest category of PFOA exposure, although there was no adjustment for confounding (Olsen et al. 1998). Another study found that mothers who were overweight or obese before pregnancy had higher plasma levels of PFOS and PFOA (Fei et al. 2007), and a third observed higher PFOS and PFOA levels in cord blood of both overweight and underweight women (Apelberg et al. 2007).

### Previous studies in animals

Unlike in humans, studies in rodents found consistent inverse associations between cholesterol levels and exposure to PFOS and PFOA, although doses administered were much higher than typical human exposure levels (Martin et al. 2007; Thibodeaux et al. 2003). In cynomolgus monkeys, decreased TC was reported as the earliest reliable measure of a clinical response after PFOS exposure (Seacat et al. 2002). This hypolipidemic effect in primates has not been seen with PFOA exposure, however (Butenhoff et al. 2002). We are not aware of similar animal studies of PFNA or PFHxS exposure.

Weight loss has also been a common finding in high-dose animal studies of PFOS and PFOA (Seacat et al. 2002; Thibodeaux et al. 2003). A recent study in mice of PFOA exposure and body weight tested a wide range of doses and looked at both adult and developmental exposure (Hines et al. 2009). Exposure during adulthood was not associated with later-life body weight effects, whereas low-dose developmental exposure led to greater weight in adulthood and increased serum leptin and insulin levels. Animals exposed to higher doses of PFOA, on the other hand, had decreased weight.

### Possible modes of action

The hypothesized mode of action for the hypolipidemic effects of PFCs in animals is through activation of PPARα, the PPAR isoform involved in lipid homeostasis and peroxisome proliferation (Wolf et al. 2008). Multiple *in vitro* studies have shown PFCs to be PPARα ligands in rodent and human cells (Vanden Heuvel et al. 2006; Wolf et al. 2008). Activation is greater as carbon backbone length increases, and carboxylates (PFOA and PFNA) have higher activation than sulfonates (PFOS and PFHxS). PFCs may also indirectly activate PPARα by interacting with fatty acid–binding proteins (Luebker et al. 2002). PPARα ligands, such as the fibrate class of cholesterol-lowering medications, inhibit secretion of cholesterol from the liver, reducing cholesterol in the serum (Kennedy et al. 2004). PPARγ is another PPAR isoform more closely involved in adipogenesis (Grun and Blumberg 2009). Some PFCs weakly activate PPARγ in certain human cell lines (Vanden Heuvel et al. 2006).

PPAR-independent mechanisms could be involved as well. PFOS and PFOA have been shown to interact with other nuclear receptors, including the constitutive activated receptor and pregnane X receptor (Ren et al. 2009).

Interspecies differences may partly explain the inconsistent cholesterol findings between animal and human studies. Humans are less sensitive to PPARα–related effects than rodents, with approximately 10-fold lower expression of PPARα in liver compared with mice (Tilton et al. 2008). There are also major differences in PFC half-life and metabolism. Whereas the half-life of PFOA in human serum is estimated to be 3.8 years, in mice it is around 18 days (Lau et al. 2007). Finally, rodents and humans have different plasma lipid profiles, with HDL, rather than LDL, predominating in rodents (Lima et al. 1998).

### Implications for the current study

The positive associations we observed between serum concentrations of PFOS, PFOA, and PFNA and TC are consistent with much of the occupational health literature regarding PFOA, even though serum concentrations in studies of workers were at least one order of magnitude higher than in NHANES. Our findings for PFOA and PFOS are also consistent with emerging results from the very large C8 Health Study cohort. Although hyperlipidemia is not consistent with the animal literature, this may be explained by differences between species and/or doses studied.

The strongest, most consistent cholesterol results were seen for PFNA, despite lower serum concentrations in the NHANES population. This is biologically plausible, given that PFC toxicity seems to increase with carbon chain length. Correlation with PFOS and/or PFOA could also partly explain the results, though PFNA is only moderately correlated with them (*r* = 0.5). Very few studies have been conducted on the possible health effects of PFNA. Another notable finding was that PFHxS consistently acted in the opposite direction of the other PFCs in the cholesterol analyses. Of the compounds studied, PFHxS has the shortest carbon chain and the longest estimated half-life. This differential effect of PFHxS is not found in the literature; more research is needed to assess possible mechanisms of PFHxS action that may differ from longer-chain PFCs.

The lack of consistent findings regarding body size is not entirely surprising. Although interesting findings have been published recently on developmental exposures in both humans and animals (Fei et al. 2007, Hines et al. 2009), adult exposures appear to be less of a concern. Effects on insulin resistance have been studied very little.

### Limitations and strengths

Our study has a number of limitations that make it exploratory in nature. The NHANES data are cross- sectional, limiting our ability to rule out reverse causality. It is possible that PFCs behave differently in the bodies of people who have higher cholesterol levels. In addition, the hypothesis has been raised that the positive associations observed here and in occupational health studies between PFCs and TC may be due to the fact that PFCs bind to β-lipoproteins and albumin in the blood (Olsen and Zobel 2007). Han et al. (2003) concluded that, in human and rat serum, more than 90% of PFOA would be bound to albumin. The only report regarding PFC binding to β-lipoproteins is a short, non-peer-reviewed document found in a U.S. Environmental Protection Agency docket that found that PFOS, PFOA, and PFHxS all bind tightly to albumin, but that differences exist in binding to β-lipoproteins, with 96% of PFOS binding compared with 64% of PFHxS and 40% of PFOA (Kerstner-Wood et al. 2003). The authors conclude that the data show “that albumin is by far the largest single protein binder for three of the four compounds tested. . . . The fourth compound, PFOS, was found to be highly bound by both albumin and β-lipoproteins.”

To address these concerns, we showed that controlling for serum albumin did not affect associations between serum PFCs and cholesterol. Confounding by PFC binding to β-lipoproteins is still an issue, although we would expect this to be most striking for PFOS, which binds most highly. The fact that we see similar results for PFOS, PFOA, and PFNA is somewhat reassuring, as is the fact that we see an inverse association with PFHxS. If major confounding by β-lipoprotein binding were occurring, we would expect to see a stronger positive association between cholesterol and PFHxS than PFOA. Our results for PFOA are also consistent with occupational studies that were able to model longitudinal data.

Additional limitations of our study include the fact that we have only one measurement of PFC and cholesterol concentrations. Because PFCs have relatively long half-lives, we can be fairly confident that blood concentrations reflect longer-term exposure, but cholesterol levels have significant variability, and multiple measures are ideal (Tietz et al. 2006). If this measurement error is random and not related to PFC level, which seems likely, it should not bias the estimate, but rather increase the standard deviation. There is also the potential for residual confounding by diet or other factors. Because NHANES measures different classes of environmental chemicals in different subsamples of the population, we were unable to consider coexposure to other chemicals suspected to disrupt weight and lipid regulation.

Despite these limitations, our study has a number of strengths. It has a relatively large sample size and the ability to account for key covariates such as alcohol consumption and use of cholesterol-lowering medications. The large population also allows for consideration of modification by age and sex. In addition to PFOA and PFOS, we examined PFNA and PFHxS, compounds that have received less scientific attention but appear important to study further.

## Conclusion

Although these results are based on cross- sectional data and are exploratory, they are consistent with much of the human epidemiologic literature and indicate that PFCs may be exerting an effect on cholesterol metabolism at environmentally relevant exposures. Our study affirms the importance of investigating PFCs other than PFOS and PFOA, particularly as industrial uses of PFOS and PFOA decline and other PFCs are substituted. PFNA may be of particular concern, as the chemical was detected in 98% of NHANES participants and serum concentrations rose between the time periods of 1999–2000 and 2003–2004 (Calafat et al. 2007). In some cases, PFNA had a greater magnitude of effect on cholesterol levels than PFOS and PFOA.

Although this study does not demonstrate a causal association between PFC exposure and serum cholesterol levels, it provides clues about where to focus future epidemiologic and toxicology research. In particular, additional studies are needed to shed light on explanations for the opposite associations with cholesterol observed for PFHxS compared with the other PFCs studied and on the relationship between PFC binding to proteins in the blood, particularly beta-lipoproteins, and cholesterol levels. Despite its limitations, this study contributes to the literature suggesting that PFC exposure may disrupt cholesterol metabolism or homeostasis in humans.

## Figures and Tables

**Table 1:** Distribution of cholesterol outcomes and PFC concentrations, persons 20–80 years of age.

	No.	Median	Mean ± SD	Range
TC (mg/dL)	860	199.0	202.1 ± 42.3	86–394
HDL (mg/dL)	860	53.0	54.6 ± 15.4	23–122
Non-HDL (mg/dL)	860	143.0	147.5 ± 43.4	52–361
LDL (mg/dL)	416	115.0	117.1 ± 35.6	21–252
PFOA (μg/L)	860	3.9	4.6 ± 3.0	0.1–37.3
Quartile 1	223	2.1	1.9 ± 0.6	0.1–2.7
Quartile 2	211	3.4	3.4 ± 0.4	2.8–3.9
Quartile 3	186	4.6	4.6 ± 0.4	4.0–5.4
Quartile 4	240	6.9	8.0 ± 3.3	5.5–37.3
PFOS (μg/L)	860	21.0	25.3 ± 20.6	1.4–392.0
Quartile 1	193	9.9	9.6 ± 2.9	1.4–13.6
Quartile 2	198	17.3	17.0 ± 1.8	13.8–19.7
Quartile 3	211	23.5	23.6 ± 2.4	19.8–28.1
Quartile 4	258	37.5	44.8 ± 28.0	28.2–392.0
PFNA (μg/L)	860	1.0	1.3 ± 1.2	0.1–10.3
Quartile 1	170	0.4	0.4 ± 0.1	0.1–0.5
Quartile 2	183	0.7	0.7 ± 0.1	0.6–0.8
Quartile 3	246	1.0	1.1 ± 0.1	0.9–1.3
Quartile 4	261	2.0	2.5 ± 1.5	1.4–10.3
PFHxS (μg/L)	860	1.8	2.6 ± 2.7	0.2–27.1
Quartile 1	217	0.8	0.7 ± 0.3	0.2–1.1
Quartile 2	239	1.5	1.5 ± 0.2	1.2–1.9
Quartile 3	233	2.4	2.6 ± 0.5	2.0–3.5
Quartile 4	171	5.3	6.7 ± 3.7	3.6–27.1

This table presents data for the population analyzed in Figure 1 and Table 2: 20- to 80-year-olds with full information on outcomes, exposures, and covariates. Quartiles of PFC exposure were calculated in the overall population (which included 12- to 19-year-olds and people missing covariate information). Therefore, the number of people in each PFC quartile is unequal.

**Table 2:** Change in cholesterol measure (milligrams per deciliter) per microgram per liter increase in PFC, persons 20–80 years of age.

	TC coefficient (95% CI)	HDL coefficient (95% CI)	Non-HDL coefficient (95% CI)	LDL coefficient (95% CI)
PFOS	0.27 (0.05 to 0.48)	0.02 (−0.05 to 0.09)	0.25 (0 to 0.50)	0.12 (−0.17 to 0.41)
PFOA	1.22 (0.04 to 2.40)	−0.12 (−0.41 to 0.16)	1.38 (0.12 to 2.65)	−0.21 (−1.91 to 1.49)
PFNA	2.01 (−1.16 to 5.18)	−0.40 (−0.90 to 0.09)	2.56 (−1.19 to 6.30)	0.50 (−3.94 to 4.93)
PFHxS	−0.93 (−1.80 to −0.06)	0.19 (−0.18 to 0.55)	−1.13 (−1.90 to −0.35)	−2.06 (−3.54 to −0.58)

All models are adjusted for age, sex, race/ethnicity, SES, saturated fat intake, exercise, time in front of a TV or computer, BMI, alcohol consumption, and smoking. We excluded values identified as influential points and outliers from the population of adults (*n* = 860) in Table 1 and Figure 1. Most analyses excluded one or two points except PFNA and TC (4), PFNA and HDL (6), PFNA and non-HDL (4), PFHxS and non-HDL (0), and PFHxs and LDL (5). See Supplemental Material, Table 4 (doi:10.1289/ehp.0901165.S1), for a full listing of the number of outliers excluded in each analysis.

## References

[b1-ehp-118-197] Apelberg BJ, Goldman LR, Calafat AM, Herbstman JB, Kuklenyik Z, Heidler J (2007). Determinants of fetal exposure to polyfluoroalkyl compounds in Baltimore, Maryland. Environ Sci Technol.

[b2-ehp-118-197] Biegel LB, Hurtt ME, Frame SR, O’Connor JC, Cook JC (2001). Mechanisms of extrahepatic tumor induction by peroxisome proliferators in male CD rats. Toxicol Sci.

[b3-ehp-118-197] Bonora E, Targher G, Alberiche M, Bonadonna RC, Saggiani F, Zenere MB (2000). Homeostasis model assessment closely mirrors the glucose clamp technique in the assessment of insulin sensitivity: studies in subjects with various degrees of glucose tolerance and insulin sensitivity. Diabetes Care.

[b4-ehp-118-197] Butenhoff J, Costa G, Elcombe C, Farrar D, Hansen K, Iwai H (2002). Toxicity of ammonium perfluorooctanoate in male cynomolgus monkeys after oral dosing for 6 months. Toxicol Sci.

[b5-ehp-118-197] Fletcher T, Savitz D, Steenland K, C8 Science Panel (2008). Status Report: Association of Perfluorooctanoic Acid (PFOA) and Perfluoroctanesulfonate (PFOS) with Lipids Among Adults in a Community with High Exposure to (PFOA). http://www.c8sciencepanel.org/pdfs/Status_Report_C8_and_lipids_Oct2008.pdf.

[b6-ehp-118-197] Calafat AM, Wong LY, Kuklenyik Z, Reidy JA, Needham LL (2007). Polyfluoroalkyl chemicals in the U.S. population: data from the National Health and Nutrition Examination Survey (NHANES) 2003–2004 and comparisons with NHANES 1999–2000. Environ Health Perspect.

[b7-ehp-118-197] CDC (Centers for Disease Control and Prevention) (2009). National Health and Nutrition Examination Survey 1999–2010 Survey Content.

[b8-ehp-118-197] CDC (Centers for Disease Control and Prevention) (2007a). MEC Laboratory Component: Plasma Glucose, Serum C-Peptide, and Insulin.

[b9-ehp-118-197] CDC (Centers for Disease Control and Prevention) (2007b). MEC Laboratory Component: Total Cholesterol and HDL-Cholesterol.

[b10-ehp-118-197] CDC (Centers for Disease Control and Prevention) (2007c). MEC Laboratory Component: Triglycerides and LDL-Cholesterol.

[b11-ehp-118-197] CDC (Centers for Disease Control and Prevention) (2007d). NHANES Mobile Exam Center Components Descriptions.

[b12-ehp-118-197] Conder JM, Hoke RA, De Wolf W, Russell MH, Buck RC (2008). Are PFCAs bioaccumulative? A critical review and comparison with regulatory criteria and persistent lipophilic compounds. Environ Sci Technol.

[b13-ehp-118-197] Costa G, Sartori S, Consonni D (2009). Thirty years of medical surveillance in perfluooctanoic acid production workers. J Occup Environ Med.

[b14-ehp-118-197] Emmett EA, Zhang H, Shofer FS, Freeman D, Rodway NV, Desai C (2006). Community exposure to perfluorooctanoate: relationships between serum levels and certain health parameters. J Occup Environ Med.

[b15-ehp-118-197] Fei C, McLaughlin JK, Tarone RE, Olsen J (2007). Perfluorinated chemicals and fetal growth: a study within the Danish National Birth Cohort. Environ Health Perspect.

[b16-ehp-118-197] Grun F, Blumberg B (2009). Endocrine disrupters as obesogens. Mol Cell Endocrinol.

[b17-ehp-118-197] Han X, Snow TA, Kemper RA, Jepson GW (2003). Binding of perfluorooctanoic acid to rat and human plasma proteins. Chem Res Toxicol.

[b18-ehp-118-197] Hines EP, White SS, Stanko JP, Gibbs-Flournoy EA, Lau C, Fenton SE (2009). Phenotypic dichotomy following developmental exposure to perfluorooctanoic acid (PFOA) in female CD-1 mice: low doses induce elevated serum leptin and insulin, and overweight in mid-life. Mol Cell Endocrinol.

[b19-ehp-118-197] Kennedy GL, Butenhoff JL, Olsen GW, O’Connor JC, Seacat AM, Perkins RG (2004). The toxicology of perfluorooctanoate. Crit Rev Toxicol.

[b20-ehp-118-197] Kerstner-Wood C, Coward L, Gorman G (2003). Protein Binding of Perfluorohexane Sulfonate, Perfluorooctane Sulfonate and Perfluorooctanoate to Plasma (Human, Rat, and Monkey), and Various Human-Derived Plasma Protein Fractions: Southern Research Institute.

[b21-ehp-118-197] Korn EL, Graubard BI (1991). Epidemiologic studies utilizing surveys: accounting for the sampling design. Am J Public Health.

[b22-ehp-118-197] Lau C, Anitole K, Hodes C, Lai D, Pfahles-Hutchens A, Seed J (2007). Perfluoroalkyl acids: a review of monitoring and toxicological findings. Toxicol Sci.

[b23-ehp-118-197] Lima VL, Sena VL, Stewart B, Owen JS, Dolphin PJ (1998). An evaluation of the marmoset *Callithrix jacchus* (sagui) as an experimental model for the dyslipoproteinemia of human Schistosomiasis mansoni. Biochim Biophys Acta.

[b24-ehp-118-197] Lin CY, Chen PC, Lin YC, Lin LY (2009). Association among serum perfluoroalkyl chemicals, glucose homeostasis, and metabolic syndrome in adolescents and adults. Diabetes Care.

[b25-ehp-118-197] Liu J, Sempos CT, Donahue RP, Dorn J, Trevisan M, Grundy SM (2006). Non-high-density lipoprotein and very-low-density lipoprotein cholesterol and their risk predictive values in coronary heart disease. Am J Cardiol.

[b26-ehp-118-197] Luebker DJ, Hansen KJ, Bass NM, Butenhoff JL, Seacat AM (2002). Interactions of fluorochemicals with rat liver fatty acid-binding protein. Toxicology.

[b27-ehp-118-197] Martin MT, Brennan RJ, Hu W, Ayanoglu E, Lau C, Ren H (2007). Toxicogenomic study of triazole fungicides and perfluoroalkyl acids in rat livers predicts toxicity and categorizes chemicals based on mechanisms of toxicity. Toxicol Sci.

[b28-ehp-118-197] Matthews DR, Hosker JP, Rudenski AS, Naylor BA, Treacher DF, Turner RC (1985). Homeostasis model assessment: insulin resistance and beta-cell function from fasting plasma glucose and insulin concentrations in man. Diabetologia.

[b29-ehp-118-197] Olsen GW, Burris JM, Burlew MM, Mandel JH (2003). Epidemiologic assessment of worker serum perfluorooctanesulfonate (PFOS) and perfluorooctanoate (PFOA) concentrations and medical surveillance examinations. J Occup Environ Med.

[b30-ehp-118-197] Olsen GW, Burris JM, Ehresman DJ, Froehlich JW, Seacat AM, Butenhoff JL (2007). Half-life of serum elimination of perfluorooctanesulfonate, perfluorohexanesulfonate, and perfluorooctanoate in retired fluorochemical production workers. Environ Health Perspect.

[b31-ehp-118-197] Olsen GW, Gilliland FD, Burlew MM, Burris JM, Mandel JS, Mandel JH (1998). An epidemiologic investigation of reproductive hormones in men with occupational exposure to perfluorooctanoic acid. J Occup Environ Med.

[b32-ehp-118-197] Olsen GW, Zobel LR (2007). Assessment of lipid, hepatic, and thyroid parameters with serum perfluorooctanoate (PFOA) concentrations in fluorochemical production workers. Int Arch Occup Environ Health.

[b33-ehp-118-197] Ramos RG, Olden K (2008). The prevalence of metabolic syndrome among US women of childbearing age. Am J Public Health.

[b34-ehp-118-197] Ren H, Vallanat B, Nelson DM, Yeung LW, Guruge KS, Lam PK (2009). Evidence for the involvement of xenobiotic-responsive nuclear receptors in transcriptional effects upon perfluoroalkyl acid exposure in diverse species. Reprod Toxicol.

[b35-ehp-118-197] Sakr CJ, Kreckmann KH, Green JW, Gillies PJ, Reynolds JL, Leonard RC (2007a). Cross-sectional study of lipids and liver enzymes related to a serum biomarker of exposure (ammonium perfluorooctanoate or APFO) as part of a general health survey in a cohort of occupationally exposed workers. J Occup Environ Med.

[b36-ehp-118-197] Sakr CJ, Leonard RC, Kreckmann KH, Slade MD, Cullen MR (2007b). Longitudinal study of serum lipids and liver enzymes in workers with occupational exposure to ammonium perfluorooctanoate. J Occup Environ Med.

[b37-ehp-118-197] Seacat AM, Thomford PJ, Hansen KJ, Olsen GW, Case MT, Butenhoff JL (2002). Subchronic toxicity studies on perfluorooctanesulfonate potassium salt in cynomolgus monkeys. Toxicol Sci.

[b38-ehp-118-197] Thibodeaux JR, Hanson RG, Rogers JM, Grey BE, Barbee BD, Richards JH (2003). Exposure to perfluorooctane sulfonate during pregnancy in rat and mouse. I: maternal and prenatal evaluations. Toxicol Sci.

[b39-ehp-118-197] Tietz NW, Burtis CA, Ashwood ER, Bruns DE (2006). Tietz Textbook of Clinical Chemistry and Molecular Diagnostics.

[b40-ehp-118-197] Tilton SC, Orner GA, Benninghoff AD, Carpenter HM, Hendricks JD, Pereira CB (2008). Genomic profiling reveals an alternate mechanism for hepatic tumor promotion by perfluorooctanoic acid in rainbow trout. Environ Health Perspect.

[b41-ehp-118-197] Vanden Heuvel JP, Thompson JT, Frame SR, Gillies PJ (2006). Differential activation of nuclear receptors by perfluorinated fatty acid analogs and natural fatty acids: a comparison of human, mouse, and rat peroxisome proliferator-activated receptor-alpha, -beta, and -gamma, liver X receptor-beta, and retinoid X receptor-alpha. Toxicol Sci.

[b42-ehp-118-197] White SS, Calafat AM, Kuklenyik Z, Villanueva L, Zehr RD, Helfant L (2007). Gestational PFOA exposure of mice is associated with altered mammary gland development in dams and female offspring. Toxicol Sci.

[b43-ehp-118-197] Wolf CJ, Takacs ML, Schmid JE, Lau C, Abbott BD (2008). Activation of mouse and human peroxisome proliferator-activated receptor alpha by perfluoroalkyl acids of different functional groups and chain lengths. Toxicol Sci.

